# Supplementary material for: Prenatal Cases Reflect the Complexity of the COL1A1/2 Associated Osteogenesis Imperfecta
Source: Genes (Basel). 2022 Sep 2;13(9):1578. doi: 10.3390/genes13091578 (PMC9498730; doi:10.3390/genes13091578)
Supplement: Supplementary file 1 [file genes-13-01578-s001.zip › genes-1861891-supplementary.pdf]

Supplementary Material 1

Structural and molecular dynamics analysis on the CANT1-V186I variant

Methods:

The SWISS-MODEL program (https://swissmodel.expasy.org/) was applied to model the specific segmental domains of CANT1 (Residue 85-401) containing the mutation site(V186I), using the crystal structure with X-RAY DIFFRACTION of 1.7 Å as the template[38]) with default parameters. The molecular dynamics (MD) prediction analysis was generated by GROMACS (version 2020.6)[39]. We carried out 120ns MD simulations on wild type (WT) CANT1 and V186I - CANT1 models. The CHARMM36 force field was applied to add hydrogen atoms and N-terminal and C-terminal patches to the models[40]. The wild type or the mutant structure of the protein was immersed in cubic boxes which contains water and placed at least 1.0 nm from the box edge.Na^+ and Cl^- ions were used for neutralization. The MD simulations were performed at a temperature of 300K for 60ns after energy minimization,equilibration. The following GROMACS distribution programs were used in MD trajectories: gmx rms, gmxrmsf, gmx gyrate, gmxsasa, and gmxhbond. These MD analyses generated parameters values for root-mean-square deviation (RMSD), root-mean-square fluctuation (RMSF), radius of gyration (Rg), solvent accessible surface area (SASA), and number of h-bonds.

Results:

TheCANT1^{V186I} variant impact the protein stability and secondary structure.

The final and converged models out of structure prediction are depicted in Figure (Fig. S1-A). Although CANT1^{V186I} affect whole flexibility slightly (Fig. S1-B and C). The number of hydrogen bonds in whole protein is comparable to the wild type (Fig. S1-D). Amino acid residue V186in wild type formed similar number of hydrogen bonds with the other residues compared to the variant residue V186I (Fig. S1-E). Furthermore, V186I mutation does not affected compactness (Fig. S1-F) and structureof protein significantly(Fig. S1-G).

Figure S1 and legend

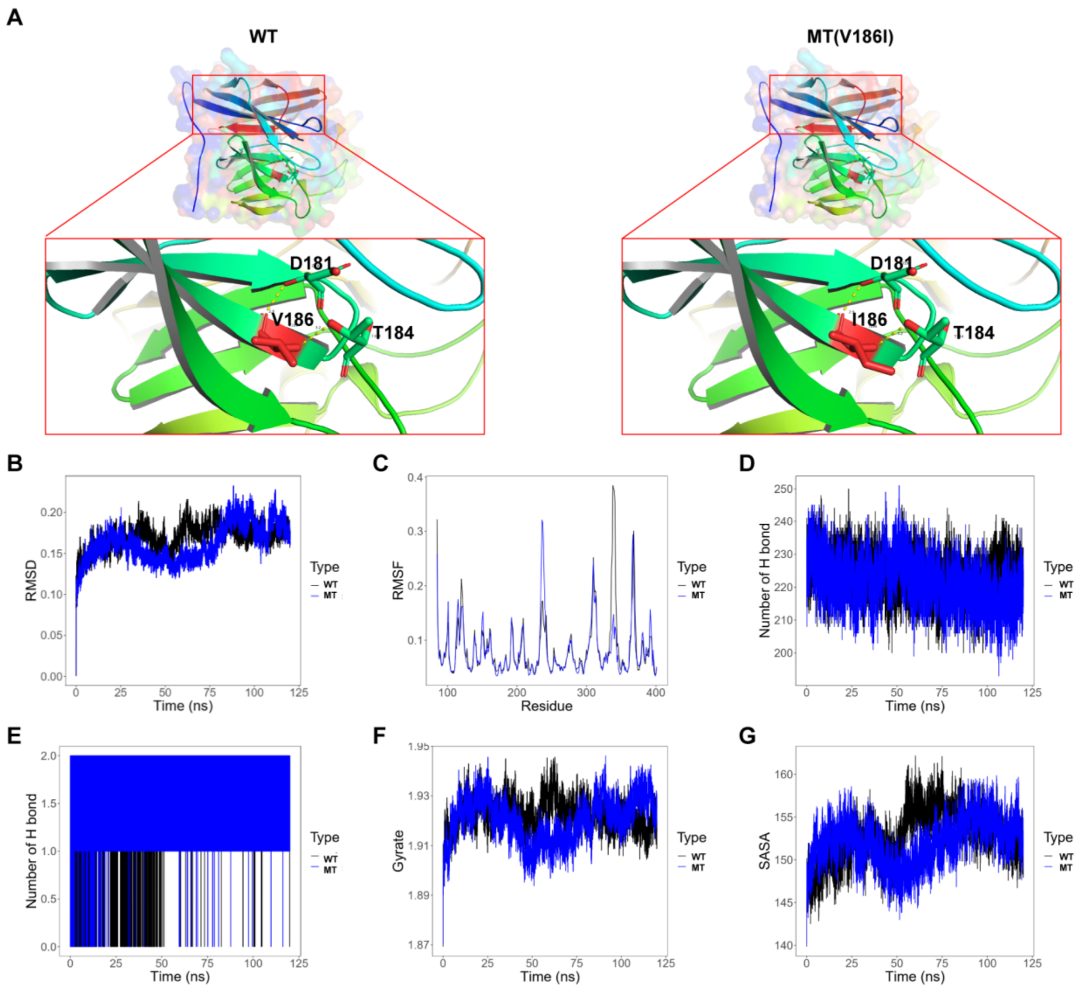

Figure S1. Structural and MD results of the CANT1^{V186I} variant.(A)The structures of the domain containing the WT and V186I models (Residues forming hydrogen bonds with the residue P65 or V186I are depicted in stick representation; Dotted yellow lines represent the hydrogen bonds). (B) RMSD: The trajectory of RMSD (Ca) for the two proteins, which compared every structure in the trajectory to the reference/initial frame (0 ns) by computing the root mean square deviation (RMSD). RMSD is a numerical measurement representing the difference between two structures. In molecular dynamics, we are interested in how structures and parts of structures change over time as compared to the starting point, so the trajectory of RMSD can be used to identify large changes in protein structure as compared to the starting point. (C) RMSF: RMSF of the two proteins calculated from each simulation, which

computed the root mean square fluctuation (RMSF) of atomic positions in the trajectory after fitting to the reference/initial frame (0 ns). RMSF is a numerical measurement similar to RMSD, but instead of indicating positional differences between entire structures over time, RMSF is a calculation of individual residue flexibility, or how much a particular residue moves (fluctuates) during a simulation. (D) H_bond_total:The number of hydrogen bonds formed in wild type or mutated protein. Although the hydrogen bond is much weaker than a covalent bond, the large number of imide and carbonyl groups in peptide chains results in the formation of numerous hydrogen bonds, and these are important for structures to stabilize the folding of the peptide backbone and facilitate molecular interactions. (E) H_bond_V186I: The number of hydrogen bonds formed between the residue V186 or V186Iand the other residues for each structure in the trajectory. (F) Gyrate: Rg analysis of wild-type CANT1 and its variants. Rg is a measurement of structural displacement of protein atoms from their common center of mass throughout the simulationand provide comprehensive information on protein compactness over time. (G) SASA: SASA analysis of wild-type CANT1 and its variants. SASA measures the exposed surface in protein structures accessible to solvent molecules. SASA analysis provides relevant information of exposure to its solvent environment over time.
